# Environmental Resistance and Fatigue Behaviors of Epoxy/Nano-Boron Nitride Thermally Conductive Structural Film Adhesive Toughened by Polyphenoxy

**DOI:** 10.3390/polym13193253

**Published:** 2021-09-24

**Authors:** Cheng’e Yue, Shaobo Dong, Ling Weng, Yazhen Wang, Liwei Zhao

**Affiliations:** 1 Heilongjiang Province Key Laboratory of Polymeric Composition Material, School of Materials Science and Engineering, Qiqihar University, Qiqihar 161006, China; yuece0219@qqhru.edu.cn (C.Y.); dongshaobo@qqhru.edu.cn (S.D.); wyz6166@163.com (Y.W.); 2 School of Materials Science and Chemical Engineering, Harbin University of Science and Technology, Harbin 150040, China; 3 Institute of Petrochemistry, Heilongjiang Academy of Sciences, Harbin 150040, China

**Keywords:** structural film adhesive, thermal conductivity, environmental resistance, fatigue behaviors, boron nitride nanosheets

## Abstract

The thermally conductive structural film adhesive not only carries large loads but also exhibits excellent heat-transfer performance, which has huge application prospects. Herein, a novel epoxy (Ep) thermally conductive structural film adhesive was prepared using polyphenoxy (PHO) as the toughening agent and film former, boron nitride (BN) nanosheets as the thermally conductive filler, and polyester fabric as the carrier. When the amount of PHO in the epoxy matrix was 30 phr and the content of nano-BN was 30 wt.% (Ep/PHO30/nBN30), the adhesive resin system showed good film-forming properties, thermal stability, and thermal conductivity. The glass transition temperature of Ep/PHO30/nBN30 was 215 °C, and the thermal conductivity was 209.5% higher than that of the pure epoxy resin. The Ep/PHO30/nBN30 film adhesive possessed excellent adhesion and peeling properties, and the double-lap shear strength at room temperature reached 36.69 MPa, which was 21.3% higher than that of pure epoxy resin. The double-lap shear strength reached 15.41 MPa at 150 °C, demonstrating excellent high temperature resistance. In addition, the Ep/PHO30/nBN30 film adhesive exhibited excellent heat-aging resistance, humidity, and medium resistance, and the shear strength retention rate after exposure to the complicated environment reached more than 90%. The structural film adhesive prepared showed excellent fatigue resistance in the dynamic load fatigue test, the double-lap shear strength still reached 35.55 MPa after 1,000,000 fatigue cycles, and the strength retention rate was 96.9%, showing excellent durability and fatigue resistance.

## 1. Introduction

The structural adhesive is an interface material that transmits large static and dynamic loads [1,2], and it is widely used in the fields of aerospace [3,4], transportation [5,6], wind power [7], and construction [8]. Adhesives also play an important role in the bonding and riveting hybrid joints used in the aerospace and transportation industries [9]. The high-temperature structural film adhesive comprises the advantages of uniform quality per unit area, convenient construction, high bonding strength, and high reliability [10,11], and it serves as the bonding of structural parts in the aerospace field, such as metal-to-metal seal s [12,13], composites [14], and the honeycomb sandwich structure [15]. Epoxy (Ep) structural adhesives are a kind of structural adhesive with a wide range of applications due to their excellent process, performance, and bonding properties [16,17,18,19]. Vietri et al. [20] developed a novel paste adhesive as the bond of composite adherents based on epoxy/nanostructured carbon fillers, in which the carbon nanofillers significantly improved the bond strength of the joints and changed their failure mode. Jee et al. [21] fabricated core–shell structured materials to encapsulate curing agents by a solvent-free method, which significantly improved the storage stability while maintaining the rapid-curing performance. Ke et al. [22] prepared a novel film adhesive for the bonding of carbon fiber reinforced polymer (CFRP) composites to steel substrates, and the results showed that there was a stronger interfacial bond in the adhesive layer and better adherence than in the intralaminar of CFRPs. The development of technology put forward functional requirements for adhesive materials, such as adhesives with thermal conductivity [23,24,25,26], wave absorption [27], and electromagnetic shielding properties [28,29]. Researchers developed a series of functional adhesives to meet the needs of different fields [30]. In addition to the ability of transferring large loads, the thermally conductive structural adhesives also transfer heat quickly within the structural parts, which keeps the heat evenly distributed in the bonded joints while dissipating heat to reduce thermal stress and improve the safety, reliability, and service life of the bonded structural parts. Hwang et al. [31] reported an epoxy-based thermally conductive adhesive using boron nitride (BN) as the filler, and the thermal conductivity of the mixture was 1.6 times higher than that of the epoxy matrix. Moriche et al. [32] developed a thermally conductive adhesive using graphene nanoplatelets as the reinforcement and thermally conductive filler; the thermal conductivity was increased by 306% with 10 wt.% of the graphene nanoplatelets, and the single-lap shear strength was not reduced. Fu et al. [33] treated micro-sized or nano-sized alumina and boron nitride with silanes or diisocyanate to investigate the effects of the surface modification and particle size on the performance of thermally conductive epoxy adhesives. At present, most studies only focus on the preparation and static mechanical properties of thermally conductive adhesives, and there are few studies on the durability, environmental resistance, and fatigue properties of the thermally conductive epoxy structural film adhesive. It is of great significance to systematically investigate the mechanical behaviors and mechanical properties of the thermally conductive epoxy structural film adhesive in different environments, as well as fatigue loads, which can offer instruction and reference for the design of the ultimate load in an actual complex environment to ensure the safety and reliability of the bonded parts in service [34].

Therefore, a thermally conductive epoxy structural film adhesive is prepared using thermoplastic-toughened epoxy resin as the matrix, BN nanosheets as the thermally conductive filler, and polyester fabric as the carrier. The rheological properties, heat resistance, and thermal conductivity of the adhesive resin system are analyzed. The bonding performance, peeling performance, environmental resistance, and fatigue performance of the thermally conductive epoxy structural film adhesive are studied, and the failure modes of the test pieces under different conditions are explored. The thermally conductive epoxy structural film adhesive is equipped with good manufacturability, the glass transition temperature reaches 215 °C, and the thermal conductivity is 0.65 W/mK, which exhibits excellent bonding performance, environmental resistance, and fatigue resistance.

## 2. Experimental Section

### 2.1. Materials

Bisphenol A epoxy resin (epoxy value = 0.51 mol/100 g), 4,4′-diaminodiphenylsulphone (DDS), and boron nitride nanosheets (BN) were purchased from Meryer Chemical (Shanghai, China). Polyphenolic resin (PHO) was obtained from Union Carbine Co. (Danbury, CT, USA). All chemicals were used without further purification.

### 2.2. Fabrication of Modified Epoxy Resin and Structural Adhesives

The formula of the modified epoxy resin and epoxy adhesive is listed in Table 1. The preparation of the PHO-modified epoxy resin was as follows: PHO and Ep with different contents were added into a constant-temperature stirrer and stirred at 180 °C for 30 min to fully mix the raw materials; then, the mixture was poured out and cooled for later use. The PHO-modified epoxy resin, curing agent DDS, and BN nanosheets were mixed in a double-roller mill to prepare the structural adhesives. The thermally conductive film adhesive was then prepared at 70 °C using a three-roll laminator, the thickness of the films was about 250 μm, and the obtained film was compounded manually with polyester carriers.

### 2.3. Preparation of Double-Lap Shear Specimens

The double-lap shear samples were prepared to characterize the mechanical properties of the structural adhesives. Aluminum plates were subjected to phosphoric acid anodization before use for surface treatment. Aluminum (2024-T3, 100 mm × 180 mm) with a thickness of 3.2 mm and 1.6 mm was used to fabricate the double-lap shear specimen, and aluminum (2A12, 50 mm × 200 mm) with a thickness of 0.3 mm and 2.0 mm was used for the fabrication of the peel specimen. The treated aluminum plates were bonded with adhesives according to Figure S1 and then cured in a constant temperature air oven at 180 °C for 3 h with an external pressure of 0.3 MPa. The cured samples were then sawn into specimens for the double-lap shear test according to Figure S2.

### 2.4. Preparation of Specimens for Peel Test

The anodized aluminum skin and aluminum plates were bonded together by the thermally conductive structural adhesives to prepare metal-to-metal peeling samples. The anodized aluminum skin and the aluminum honeycomb washed with acetone were bonded together by the film adhesive to prepare the honeycomb sandwich structure.

### 2.5. Characterizations

The dynamic viscosity and processing temperature characteristics of the structural adhesive were tested using the DHR-1 rotary rheometer (TA, Linton, UT, USA), and the temperature scanning range was 30–250 °C. The reaction characteristics of the adhesive system were studied by the differential scanning calorimetry (DSC) method (Q20, TA, Linton, UT, USA). Approximately 10 mg of samples were added to the aluminum crucible with a lid and then put into the equipment for testing, and the temperature scanning range was 30–300 °C. The heat capacity of the cured adhesive was obtained by the DSC method, using sapphire as the standard sample, and the temperature scanning range was −20–60 °C. The glass transition temperature (T_g_) values were determined using the dynamic mechanical analysis (DMA) method from room temperature to 300 °C. The double cantilever beam mode was used, and the size of the specimen was cut to 50 mm × 10 mm × 3 mm. The temperature sweep range was 25 to 300 °C, the heating rate was 5 °C/min, and the frequency was fixed at 1 Hz. The laser thermal conductivity meter (Netzsch LFA 457 Microflash™, Selb, Germany) was used to measure the thermal diffusivity of the nanocomposites. The sample was prepared into a disc with a diameter of 12.7 mm and a thickness of approximately 1.5 mm. The thermal conductivity was then calculated according to the following Equation (1):

(1) $$\lambda =\alpha C_{p}\rho$$

where λ denotes the thermal conductivity, *α* represents the thermal diffusivity, C_p_ expresses the heat capacity determined by DSC, and *ρ* is the density measured with a densimeter. The microscopic morphology of the BN nanosheets was observed using a JEM 2000 FX II transmission electron microscope (TEM) (Jeol, Akishima, Tokyo, Japan). The microscopic morphologies of the cured adhesives were observed using a scanning electron microscope (Quanta FEG250, FEI, Hillsboro, OR, USA). The double-lap shear strength data were collected using a universal stretching machine (Instron 8801, Norwood, MA, USA) with a stretching speed of 9 MPa/min based on ASTM D3528. Each group was tested at least 5 times, and all reported tensile property data were the averages of 5 tests. For the thermal aging, and thermal and humid tests, the samples exposed to the test environment were taken out and then cooled to room temperature, and the shear test was performed at room temperature to obtain the shear strength. For the medium resistance test, the samples were taken out from the medium and wiped, and the shear test was carried out at room temperature. The metal-to-metal peel test and honeycomb sandwich peel test were carried out on a universal testing machine (Instron 5969, Norwood, MA, USA), and the 90° peeling method was adopted with a speed of 100 mm/min. The dynamic fatigue tests were conducted on a universal testing machine with a frequency of 30 Hz, and the change in the dynamic load was set in a sinusoidal manner by the program. The cycle test times were 10^2^, 10^3^, 10^4^, 10^5^, and 10^6^, and then the specimen after the fatigue test was stretched until it failed.

## 3. Results and Discussion

### 3.1. Rheological Analysis

**Figure 1:**
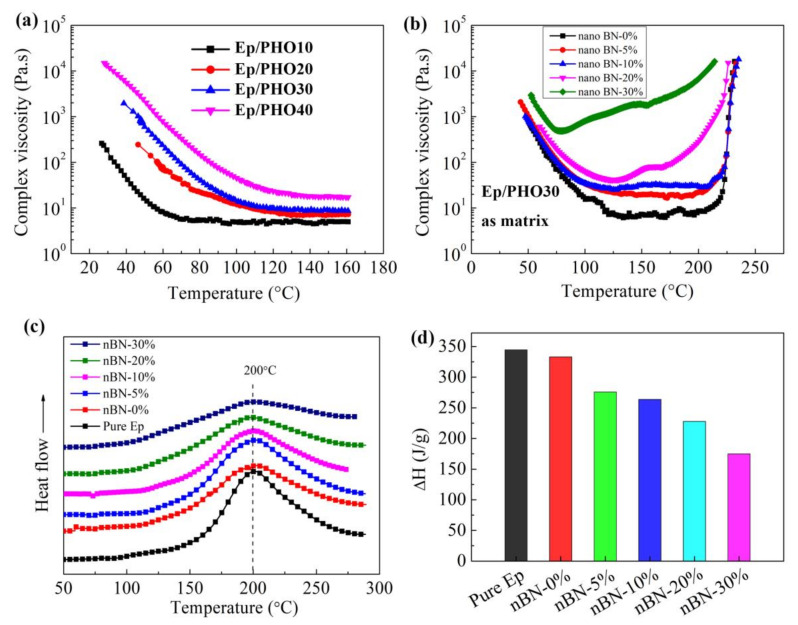
(**a**) Viscosity–temperature curves of Ep/PHO blends, (**b**) viscosity–temperature curves of the thermally conductive adhesive systems, (**c**) DSC curves of the adhesive systems, (**d**) the reaction enthalpy of the adhesive systems.

The dynamic rheological properties of the epoxy/PHO blends and the BN nanosheet-modified epoxy structural adhesives were analyzed using rheological methods. The processing temperature of the epoxy structural film adhesive at 50–80 °C was the most suitable temperature for solvent-free preparation of structural film adhesive. The addition of PHO increased the complex viscosity of the epoxy/PHO blend as shown in Figure 1a. In the temperature range of 50–80 °C, the viscosity of the epoxy/PHO blend was low when the PHO content was low, while that of the epoxy/PHO blend was too high to process when the PHO content was increased to 40 phr, which indicated that these situations were not conducive to the preparation of epoxy structural film adhesive. Figure 1b shows the viscosity–temperature curves of the epoxy structural adhesives with different contents of BN nanosheets. Although the increase in the content of BN nanosheets improved the complex viscosity of the adhesive to a certain extent, the viscosity in the range of 50–80 °C is also suitable for the preparation of film adhesive. In addition, the curing behaviors of the adhesive system were not significantly changed. As shown in Figure 1c, the reaction peak temperature of all modified adhesive systems is about 200 °C, which is unchanged compared with the pure epoxy resin. The shape of the DSC curve is almost the same, except that the reaction enthalpy becomes smaller with the increase in BN content (Figure 1d). This is due to the reduction in the relative content of epoxy resin. The reaction process of the modified adhesive still followed the curing conditions of the epoxy/DDS system.

### 3.2. Thermal Stability and Thermal Conductivity of Structural Adhesives

**Figure 2:**
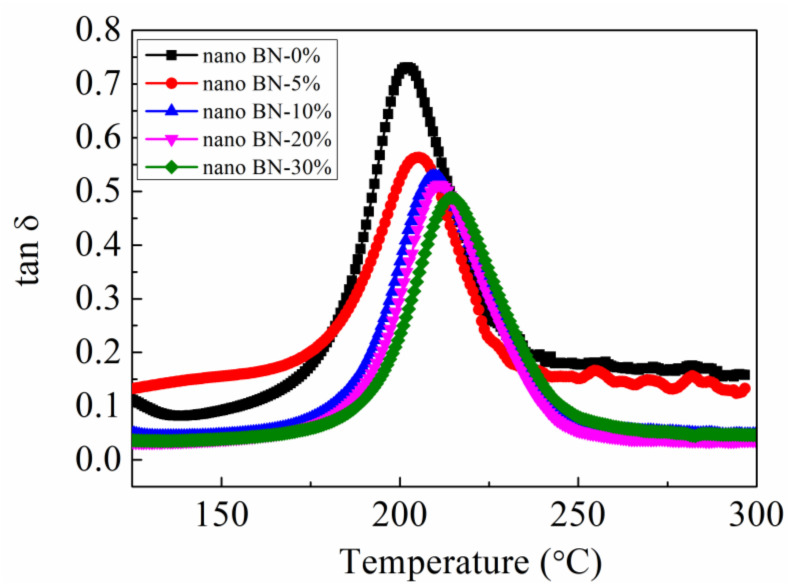
DMA curves of Ep/PHO30 thermally conductive adhesive systems.

**Figure 3:**
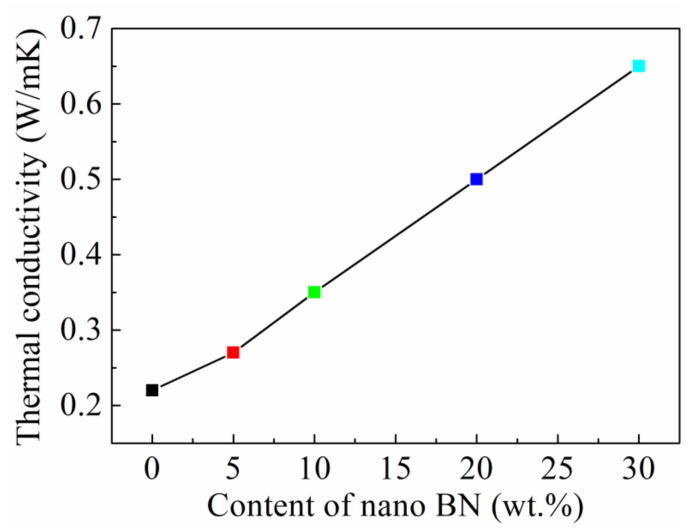
Thermal conductivity of Ep/PHO30 thermally conductive adhesive system.

The glass transition temperature (T_g_) is an important indicator for the maximum working temperature of the structural adhesives. The DMA test was used to characterize the effects of the BN nanosheets on the T_g_ of the adhesives, as shown in Figure 2. The T_g_ of the modified adhesive system increased from 202 to 215 °C in response to the increase in the content of BN nanosheets, indicating that the BN nanosheets improved the high-temperature performance of the structural adhesives. In addition, the addition of BN nanosheets effectively improved the thermal conductivity of the modified structural adhesive, as shown in Figure 3. The thermal conductivity was calculated according to Formula (1), and the heat capacity curves and the corresponding parameters are shown in Figure S3 and Table S1, respectively. The thermal conductivity of the modified adhesive increased from 0.21 to 0.65 W/mK as the content of the BN nanosheets increased, which was increased by 209.5% compared with the unmodified adhesive. The significant increase in the thermal conductivity not only enabled the structural adhesive to transfer larger static or dynamic loads when used as the interface material but also greatly enhanced the thermal conductivity of the bonding system. The effective heat dissipation reduced the thermal stress caused by the uneven thermal field, thereby improving the durability of the bonded joint.

### 3.3. Micromorphologies of BN Nanosheets and Structural Adhesive Systems

**Figure 4:**
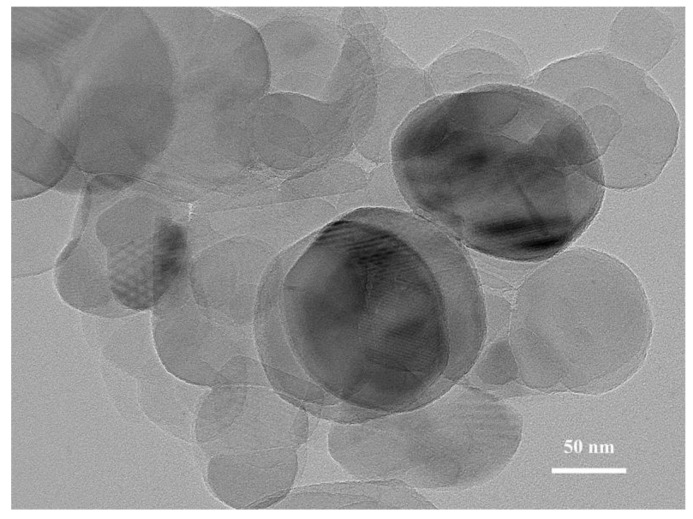
TEM image of BN nanosheets.

The microstructure of the BN nanosheets was analyzed by TEM, as shown in Figure 4. The BN nanosheets presented a typical thin disc shape with a diameter of 50–100 nm. Moreover, the nano-BN with a small thickness could effectively improve the thermal conductivity of the adhesives.

**Figure 5:**
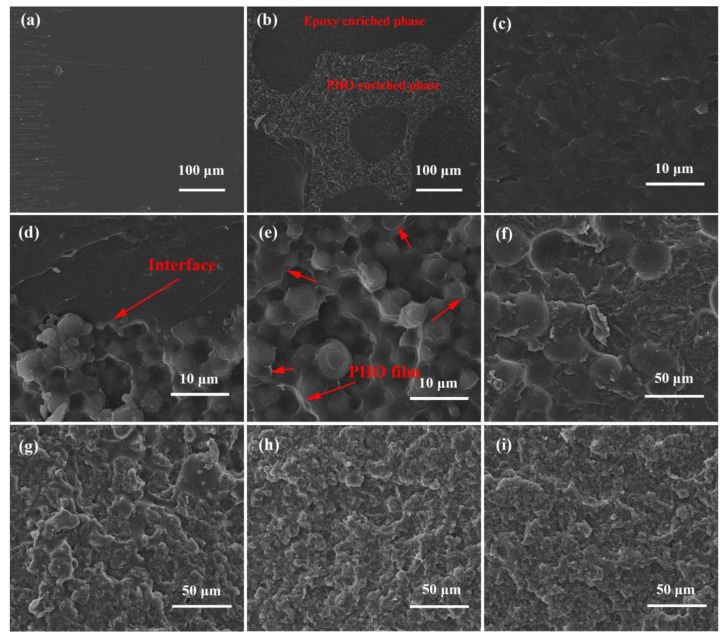
SEM images of different adhesive systems: (**a**) pure epoxy resin; (**b**) Ep/PHO30; (**c**) PHO-enriched phase in Ep/PHO30; (**d**) epoxy resin-enriched phase in Ep/PHO30; and (**e**) interface between two phases in Ep/PHO30, (**f**) Ep/PHO30/nBN5, (**g**) Ep/PHO30/nBN10, (**h**) Ep/PHO30/nBN20, and (**i**) Ep/PHO30/nBN30.

The micromorphologies of the sections of the modified structural adhesives were analyzed by SEM, and the results are shown in Figure 5. The section of the pure epoxy resin was flat and smooth, indicating its high brittleness (Figure 5a). The EP/PHO30 system exhibited a typical two-phase structure, including the epoxy-rich phase and PHO-rich phase (Figure 5b). The epoxy resin-enriched phase presented ductile fracture morphology (Figure 5c), and there were well-bonded interfaces between the two phases (Figure 5d), which had the benefit of improving the toughness of the adhesives. The continuous PHO thin film enveloped the cured spherical epoxy resin in the PHO-rich phase (Figure 5e). The two-phase structure was destroyed as the content of the BN nanosheets increased (Figure 5f,g). When the content of the BN nanosheets was equal to or higher than 20%, the two-phase structure disappeared and was replaced by a homogeneous structure (Figure 5h,i), which showed that the BN nanosheets were conducive to the blending between PHO and epoxy under the action of mechanical force.

### 3.4. Double-Lap Shear and Peel Properties of Structural Adhesives

**Figure 6:**
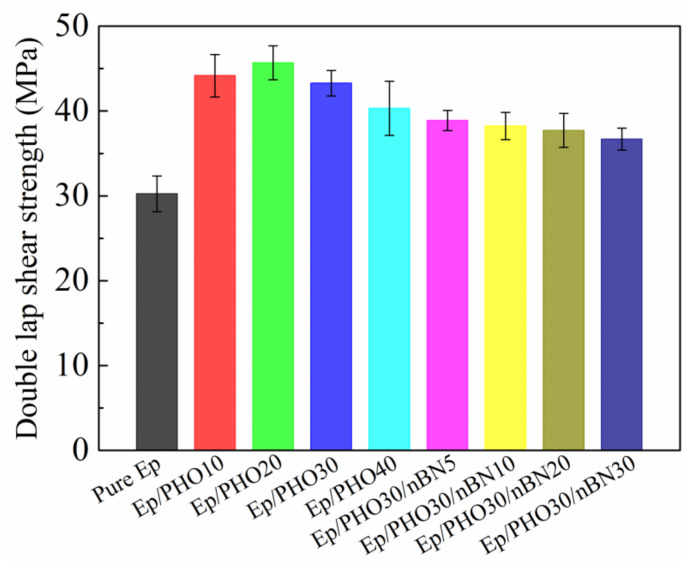
Double-lap shear strength of different adhesive systems.

The shear properties of different adhesive systems were investigated using the double-lap shear method, and the results are shown in Figure 6. The double-lap shear strength of the pure epoxy resin adhesive was 30.24 MPa, and that of the PHO-modified adhesives first increased and then decreased with the increase in PHO content. The shear strength of the Ep/PHO20 and Ep/PHO30 adhesives was 45.68 and 43.28 MPa, which was 51% and 43% higher than that of the pure epoxy resin, respectively, indicating that the PHO effectively improved the shear strength of the adhesives. The Ep/PHO30 system was selected as the adhesive matrix resin by weighing the bonding and film-forming process and performance. The BN nanosheets with different contents were added to the Ep/PHO30 system for the preparation of the film adhesive. The BN nanosheets reduced the shear strength of the modified structural film adhesive, which was due to the reduction in the toughness of the modified system caused by the rigid fillers. However, the shear strength of the film adhesive still reached 36.69 MPa when the content of the BN nanosheets was 30%, which was 21.3% higher than that of the pure epoxy resin, which shows excellent bonding performance.

**Figure 7:**
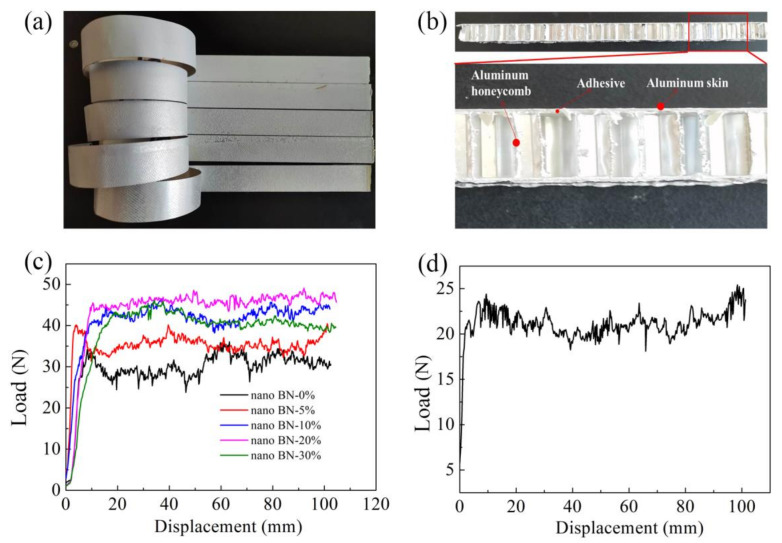
Peel performance of adhesive system: (**a**) metal-to-metal peel samples, (**b**) honeycomb sandwich structure, (**c**) metal-to-metal peel performance of Ep/PHO30 systems, and (**d**) aluminum honeycomb peel performance of Ep/PHO30/nBN30 adhesive.

The metal-to-metal peeling performance of the modified adhesive and the peeling performance of the aluminum honeycomb sandwich structure were further analyzed, and the photographs of samples and results are shown in Figure 7. The metal-to-metal peel strength first increased and then decreased with the increase in the BN nanosheets, and that of the Ep/PHO30/nBN20 adhesive was the highest, reaching 46.2 N. The average peel force of the Ep/PHO30/nBN30 adhesive was 40.5 N, which was 32.8% higher than that of the Ep/PHO30/nBN0 adhesive (30.5 N). The average peel force of the honeycomb sandwich structure bonded with the Ep/PHO30/nBN30 adhesive reached 22.4 N. The results showed that the one film adhesive can be used for the manufacture of metal-to-metal bonding structures and honeycomb sandwich structures.

### 3.5. Environmental Resistance of Structural Adhesives

**Figure 8:**
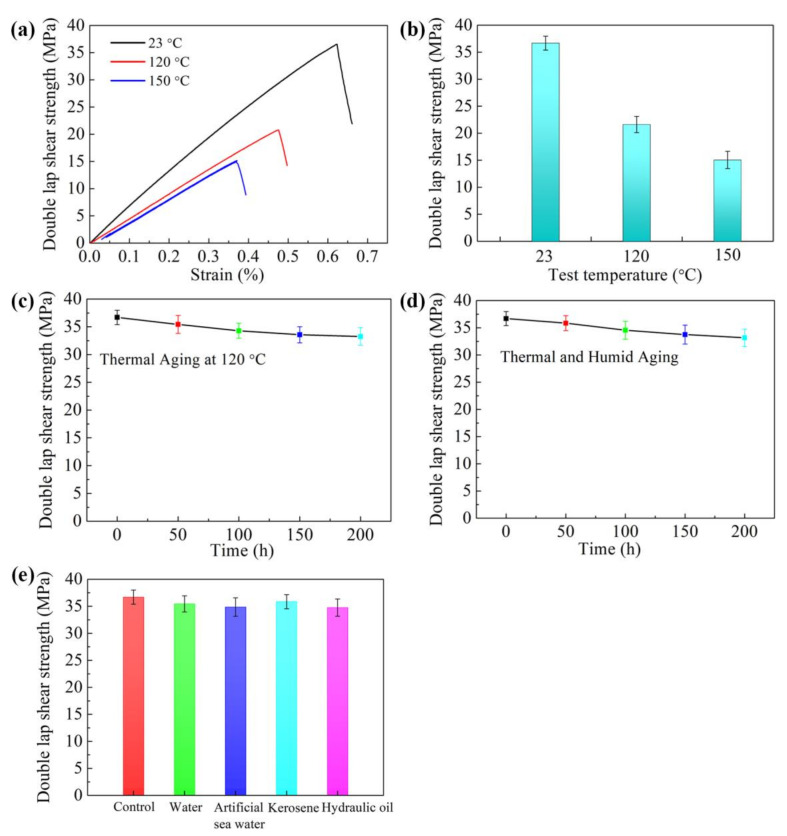
Durability performance of Ep/PHO30/nBN30 structural film adhesive: (**a**) stress–strain curves at different temperatures, (**b**) shear strength at different test temperatures, (**c**) shear strength after thermal aging at 120 °C, (**d**) shear strength after thermal and humid aging (71 °C, 95% relative humidity), and (**e**) medium resistance performance (immersed in medium for 7 days).

The above results showed that the Ep/PHO30/nBN30 adhesive system possessed excellent processing properties, thermal stability, thermal conductivity, and bonding properties. Therefore, the film adhesive based on the Ep/PHO30/nBN30 system had a wide range of application prospects. The environmental resistance performance of the Ep/PHO30/nBN30 film adhesive was further studied, and the results are shown in Figure 8. The detailed stress–strain curves of the Ep/PHO30/nBN30 film adhesive at different temperatures for double-lap shear specimens are shown in Figure 8a. The shear strength of the Ep/PHO30/nBN30 film adhesive at 23 °C was 36.69 MPa (Figure 8b), and that of the film adhesive at 120 and 150 °C decreased to 21.52 and 15.41 MPa, which indicated that the thermal conductive structural film adhesive can still be used at high temperatures.

The thermal aging performance, thermal and humid aging properties, and media resistance of the film adhesive were explored. The shear strength of the Ep/PHO30/nBN30 adhesive system was 33.25 MPa after aging at 120 °C for 200 h (Figure 8c), which was only reduced by 9.4%, indicating that the BN nanosheets were helpful in improving the heat resistance and thermal stability of the adhesives. The shear strength was 33.16 MPa after aging for 200 h in a hot and humid environment (Figure 8d), and the decline rate was only 9.7%, which indicated that the BN nanosheets prevented the diffusion of water in the adhesive. The shear strength after being immersed in water, artificial sea water, kerosene, and hydraulic oil for 7 days was 35.44, 34.85, 35.85, and 34.76 MPa, respectively, and the drop rate was less than 6% (Figure 8e). The results indicated that the thermally conductive structural adhesive had excellent environmental resistance, which can be applied in complex environments and maintain the stability of the bonded structure.

### 3.6. Fatigue Performance of Structural Film Adhesive

**Figure 9:**
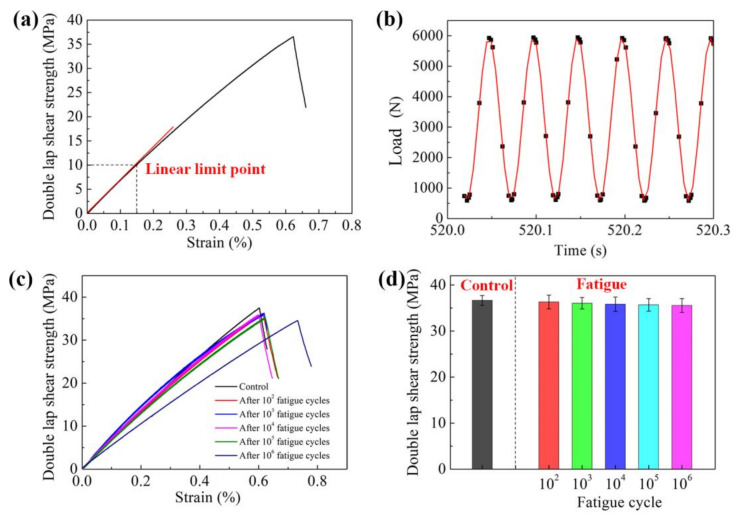
Fatigue performance of Ep/PHO30/nBN30 structural film adhesive: (**a**) stress–strain curve at room temperature, (**b**) dynamic load loading mode, (**c**) stress–strain curves after different fatigue tests, and (**d**) residual shear strength after fatigue tests.

The fatigue test of the bonded structure under dynamic loads was employed to study and analyze the influence of the dynamic loads on the bonding quality and reliability; the photo of the fatigue test equipment is shown in Figure S4. The stress–strain curve of the double-lap shear specimen is shown in Figure 9a, which shows that the adhesive underwent an elastic deformation under low strain conditions. The tangent line was drawn at the starting point of the stress–strain curve, and the point where the tangent just left the stress–strain curve was defined as the linear limit point. The linear limit point of the Ep/PHO30/nBN30 adhesive was 10 MPa. Therefore, the maximum value of the dynamic load was set to the stress value of the linear limit point (10 MPa) in the fatigue test, the minimum value was set as 1 MPa, and the dynamic load was applied in a sinusoidal mode (Figure 9b). The stress–strain curves of the double-lap shear specimens of the Ep/PHO30/nBN30 adhesive after different dynamic fatigue cycles are shown in Figure 9c. The tensile behaviors of the bonded joint did not change significantly. The strain value increased with the increase in the fatigue cycles, and the shear strength showed an insignificant decrease. The double-lap shear strength still reached 35.55 MPa after 10^6^ fatigue cycles, and the strength retention rate was 96.9%, indicating that the thermally conductive structural adhesive had excellent fatigue resistance and could ensure stability and reliability of the bonded structure under dynamic loads.

**Figure 10:**
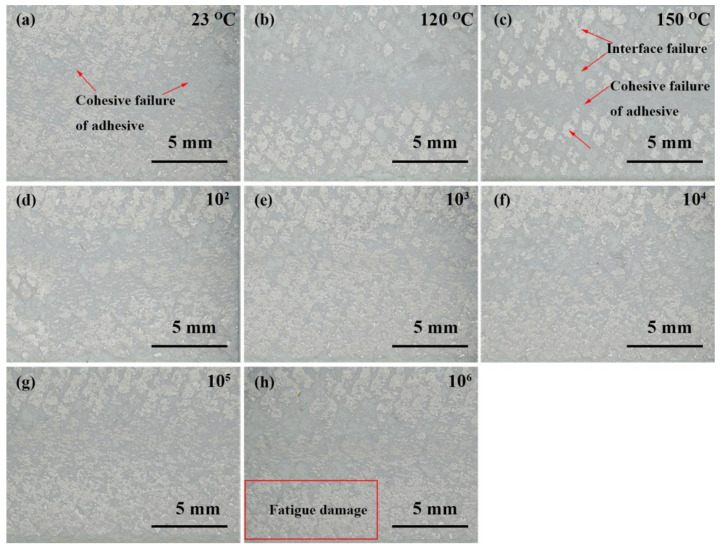
The failure mode of Ep/PHO30/nBN30 film adhesive under different test conditions: (**a**) 23 °C, (**b**) 120 °C, (**c**) 150 °C, (**d**) after 10^2^ fatigue cycles, (**e**) after 10^3^ fatigue cycles, (**f**) after 10^4^ fatigue cycles, (**g**) after 10^5^ fatigue cycles, and (**h**) after 10^6^ fatigue cycles.

The photographs of the samples after failure under different conditions are shown in Figure S5, and the optical micrographs of the failed bonding surface are shown in Figure 10. The failure mode of the sample at 23 °C was dominated by the cohesive failure of the adhesive (Figure 10a). The specimens underwent simultaneously cohesive failure and interface failure at high temperatures. The interface failure occurred at 120 °C (Figure 10b), and the interface failure ratio of the bonding interface further increased at 150 °C (Figure 10c), which was due to the reduction in the cohesive strength and interfacial strength of the adhesive at high temperatures, resulting in the decrease in the bonding strength. There was no significant difference in the failure mode of the specimens when the fatigue number was less than or equal to 100,000, the cohesive failure of the adhesive played a leading role (Figure 10d–g), and the shear strength was hardly reduced. The sample was still dominated by the cohesive failure of the adhesive after 1,000,000 fatigue cycles, but there was a certain degree of fatigue damage area (Figure 10h), which resulted in a shear strength reduction rate of 3.5%. The excellent fatigue resistance of the thermally conductive adhesive system mainly benefited from the outstanding bulk strength of the modified adhesive system and the good wettability of the modified adhesive to the bonded substrate, forming a good bonding interface.

## 4. Conclusions

In conclusion, the novel epoxy-based thermally conductive structural film adhesive was prepared, and the heat resistance, thermal conductivity, adhesive properties, environmental resistance, and fatigue behaviors were systematically investigated. PHO was employed to improve the toughness of the epoxy resins and served as an excellent film former. The BN nanosheets improved the thermal conductivity, heat resistance, and environmental resistance of the adhesive. The glass transition temperature of the modified matrix was 215 °C, and the thermal conductivity was increased by 209.5%. The prepared film adhesive showed excellent bonding and peeling properties, and the double-lap shear strength reached 36.69 MPa at room temperature, which was 21.3% higher than that of the pure epoxy resin. In addition, the structural film adhesive exhibited excellent thermal aging resistance, humid aging resistance, and medium resistance, and the shear strength retention rate after exposure to the complicated environment reached more than 90%. More importantly, the structural film adhesive was equipped with outstanding fatigue resistance in the dynamic load fatigue tests, and the failure mode of the sample was still in cohesive failure after the fatigue test. The double-lap shear strength still reached 35.55 MPa after 1,000,000 fatigue cycles, and the strength retention rate was 96.9%. The excellent performance of the prepared film adhesive was mainly due to the outstanding cohesive strength of the adhesive and the good interface formed between the adhesive and the substrate.

## Figures and Tables

**Table 1:** Formula-modified epoxy resin and epoxy adhesive.

Sample	Ep (phr)	PHO (phr)	DDS (phr)	BN Nanosheets (wt.%)
Pure Ep	100	-	30	-
Ep/PHO10	100	10	-	-
Ep/PHO20	100	20	-	-
Ep/PHO30	100	30	-	-
Ep/PHO40	100	40	-	-
Ep/PHO30/nBN5	100	30	30	5
Ep/PHO30/nBN10	100	30	30	10
Ep/PHO30/nBN20	100	30	30	20
Ep/PHO30/nBN30	100	30	30	30

## Data Availability

The data presented in this study are available on request from the corresponding author.

## Supplementary Materials

The following are available online at https://www.mdpi.com/article/10.3390/polym13193253/s1, Figure S1: Schematic diagram of the adhesive bonded aluminum plates; Figure S2: Size of double-lap shear specimen; Figure S3: The heat capacity curve of the modified adhesive system; Figure S4: The fatigue test equipment; Figure S5: Photos of failed samples; and Table S1: Heat capacity, thermal diffusivity, density, and thermal conductivity of the modified adhesive system.
