# Supplementary material for: Environmental Resistance and Fatigue Behaviors of Epoxy/Nano-Boron Nitride Thermally Conductive Structural Film Adhesive Toughened by Polyphenoxy
Source: Polymers (Basel). 2021 Sep 24;13(19):3253. doi: 10.3390/polym13193253 (PMC8513045; doi:10.3390/polym13193253)
Supplement: Supplementary file 1 [file polymers-13-03253-s001.zip › polymers-1381668-supplementary.pdf]

# *Supplementary materials*

## Environmental resistance and fatigue behaviors of epoxy/nano-boron nitride thermally conductive structural film adhesive toughened by polyphenoxy

**Cheng'e Yue [1, 2], Shaobo Dong [1], Ling Weng [2,*], Yazhen Wang[1] and Liwei Zhao [3,*]**

[1] Heilongjiang Province Key Laboratory of Polymeric Composition Material, School of Materials Science and Engineering, Qiqihar University, Qiqihar 161006, P.R.; yuece0219@qqhru.edu.cn (C. Yue); dongshaobo@qqhru.edu.cn (S. Dong); wyz6166@163.com (Y. Wang)

[2] School of Materials Science and Chemical Engineering, Harbin University of Science and Technology, Harbin 150040, P.R. China; wengling79@163.com (L. Weng)

[3] Institute of Petrochemistry, Heilongjiang Academy of Sciences, Harbin 150040, China; zhaoliwei0130@163.com; zhaoliwei0130@hit.edu.cn (L. Zhao)

***** Correspondence: wengling79@163.com (L. Weng); zhaoliwei0130@163.com and zhaoliwei0130@hit.edu.cn (L. Zhao)

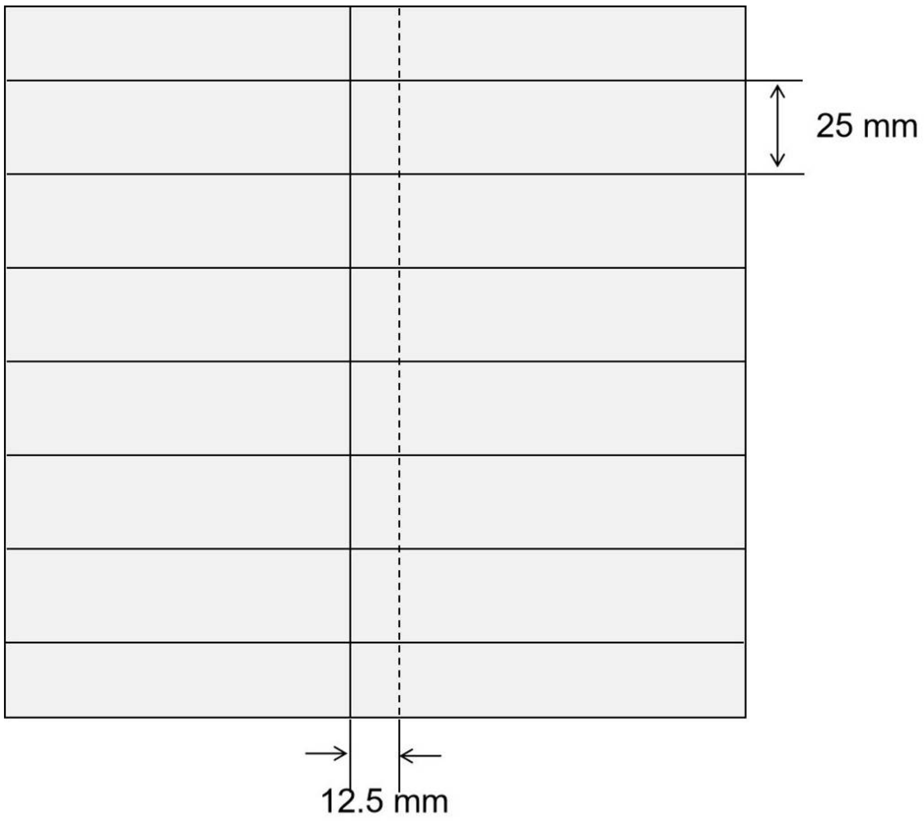


Figure S1. Schematic diagram of the adhesive bonded aluminum plates.

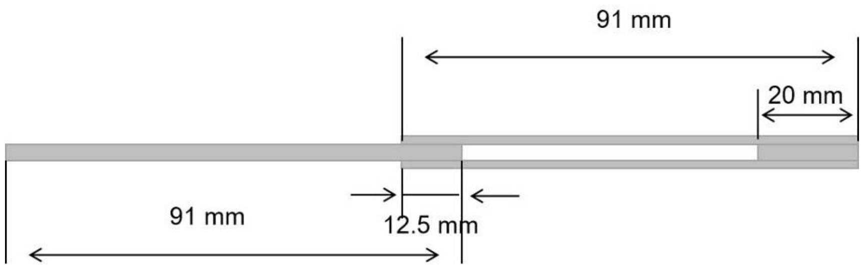


Figure S2. Size of double-lap shear specimen.

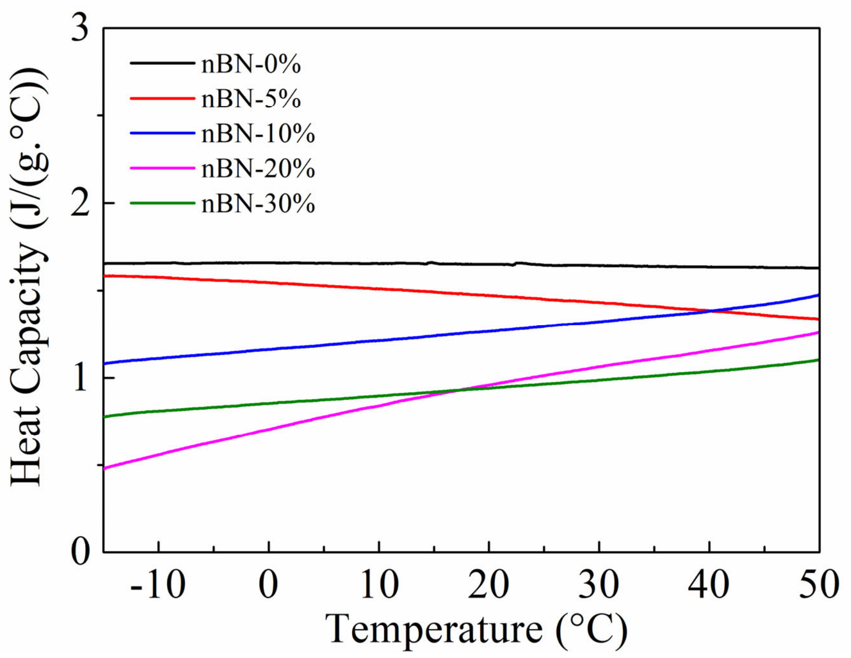


Figure S3. The heat capacity curve of the modified adhesive system.

Table S1. Heat capacity, thermal diffusivity, density and thermal conductivity of the modified adhesive system.

| Sample | $C_p$ (J/(g °C)) | α ($cm^2$/s) | ρ (g/$cm^3$) | κ (W/mK) |
|---|---|---|---|---|
| nBN-0% | 1.644 | 0.0010 | 1.30 | 0.22 |
| nBN-5% | 1.451 | 0.0014 | 1.33 | 0.27 |
| nBN-10% | 1.291 | 0.0020 | 1.35 | 0.35 |
| nBN-20% | 1.009 | 0.0035 | 1.40 | 0.50 |
| nBN-30% | 0.963 | 0.0047 | 1.44 | 0.65 |

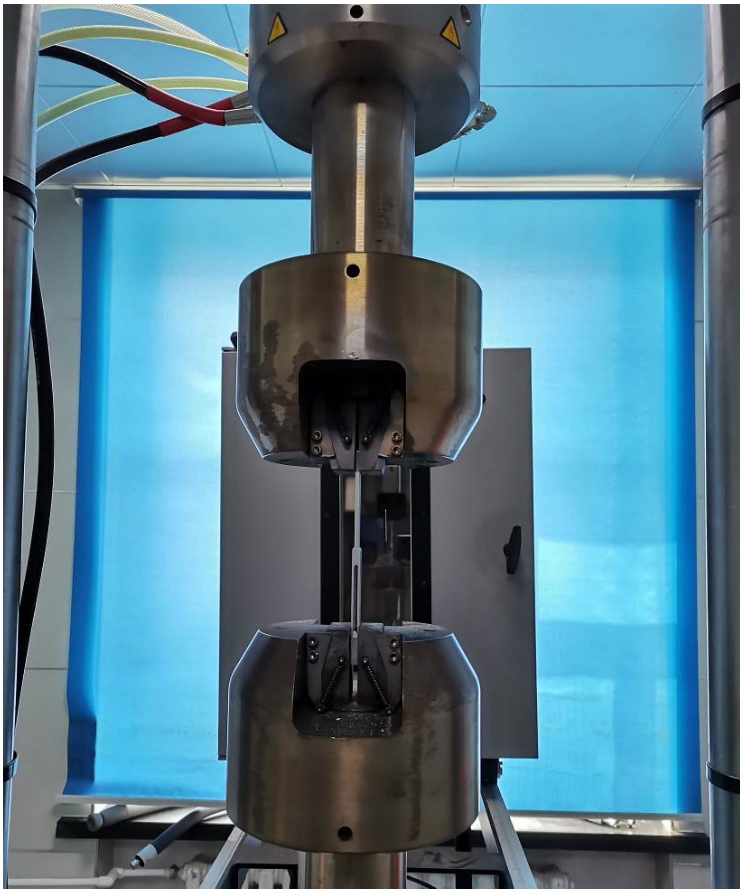

Figure S4. The fatigue test equipment.

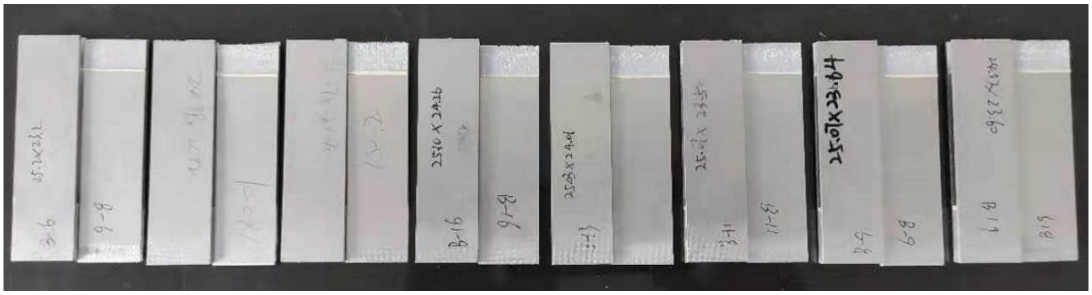

Figure S5. Photos of failed samples.
